# Vulnerability to human immunodeficiency virus infection and associated factors among married women in northwest Ethiopia: a cross-sectional study

**DOI:** 10.4069/kjwhn.2022.12.02

**Published:** 2022-12-29

**Authors:** Asiya Hussien, Abdissa Boka, Asnake Fantu

**Affiliations:** 1 College of Health Sciences, Ambo University, Ambo, Ethiopia; 2 College of Health Sciences, Addis Ababa University, Addis Ababa, Ethiopia; 3 College of Health Sciences, Ethiopian Defense University, Bishoftu, Ethiopia

**Keywords:** Acquired immunodeficiency syndrome, HIV, Marriage, Sexually transmitted diseases, Social vulnerability

## Abstract

**Purpose:**

This study investigated the vulnerability to human immunodeficiency virus (HIV) infection and associated factors among married women in northwest Ethiopia.

**Methods:**

A community-based cross-sectional survey (n=657) was conducted from April 1 to 15, 2020, in Metema District, northwest Ethiopia, in four randomly selected *kebele* administrations (the lowest level of local government). The inclusion criteria were married women aged ≥18 years residing with their husbands. Logistic regression analysis was conducted to identify factors associated with married women’s vulnerability to HIV infection.

**Result:**

Participants were on average 33.70±9.50 years and nearly one-fourth (n=148, 22.5%) were identified as vulnerable to HIV infection (i.e., experienced sexually transmitted disease symptoms or an extramarital affair of either spouse within the past 12 months). Only 18.9% reported sexual communication with their husband. Respondents who did not discuss the risk of HIV infection with their husbands had fivefold odds of vulnerability (adjusted odds ratio [AOR], 5.02; 95% confidence interval [CI], 1.43–17.5). Those who did not have premarital sex (AOR, 0.20; 95% CI, 0.05–0.77) had no worries about HIV infection (AOR, 0.27; 95% CI, 0.08–0.94), sufficient income (AOR, 0.56; 95% CI, 0.16–0.86), and less than four children (AOR, 0.69; 95% CI, 0.50–0.97) had decreased odds of being vulnerable to HIV than their counterparts.

**Conclusion:**

Not discussing risk of HIV infection with husband was a major factor of vulnerability to HIV infection as was premarital sex, worry about HIV, income, and number of children. Measures to strengthen couple’s sexual communication and support economical stability is important for decreasing HIV vulnerability.

## Introduction

Human immunodeficiency virus (HIV)/acquired immune deficiency syndrome (AIDS) has profound social, economic, and health consequences and constitutes one of the world’s most serious health and development challenges. As a leading cause of death worldwide [1,2], the HIV epidemic resulted in 1.5 million new infections globally in 2021, adding up to a total of 38.4 million people living with HIV at the end of 2021. More than half of all people living with HIV are women [3,4].

According to the World Health Organization, 25.7 million people live with HIV in Sub-Saharan Africa, with 1.1 million new infections in 2018, and this region accounts for two-thirds of the HIV burden worldwide [4-6]. The female-to-male ratio of new HIV infections ranges from 1.22:1 in West and East Africa to 1.33:1 in Southern Africa, indicating the need to respond to women’s increasing vulnerability [7,8]. In Ethiopia, the prevalence of HIV among adults is estimated at 0.9%: 1.2% among women versus 0.6% among men [9].

The vulnerability of women and girls to HIV remains elevated in Sub-Saharan Africa, as 76% of all women in the world living with HIV reside in this region [5,7,10]. In addition, Ethiopia has approximately 612,925 people living with HIV, more than half of whom are female, and the Amhara region has the third-largest number of HIV-infected people [11].

Beyond a greater physiological susceptibility toward HIV infection, women are especially vulnerable due to their disadvantages in sociological, legal, and economic factors [12]. Vulnerability to HIV infection depends on factors that influence the risk of exposure to the virus, such as the frequency of changing partners and sexual intercourse with an infected partner, and factors that affect the risk of transmission of the virus, such as condom use or the presence of another sexually transmitted infection (STI) [13-16], biological risk factors [10,13], and socioeconomic, behavioral, and structural vulnerabilities [10,11,16-18].

A study conducted in Nigeria to assess the influence of marital status and other correlates of HIV infection showed that HIV prevalence among married women (5.9%) was greater than among those who had never been married (3.4%) [19]. The power differences between women and men and gender inequality often give men more power to decide on the timing and conditions of sex and the means of preventing infection, thereby limiting women’s ability to negotiate protection with their partners [2,20]. Women's relatively weak negotiating power within marriage, as well as their limited ability to find social and economic support outside of marriage, makes it difficult for wives to stop their partners from having sex with others or engaging in extramarital sexual activities, and they cannot insist on protective measures like condom use with their spouses [13,21-25]. According to the Central Statistical Agency and ICF, the prevalence of HIV in adults is estimated at 0.9% (1.2% among women vs. 0.6% among men) [9]. Data on HIV infection patterns in India revealed that 90% of women were infected within long-term relationships or marriages [26]. Although both husbands and wives are at risk of contracting HIV from their spouses, cultural, social, and biological gender differences render women particularly vulnerable to transmission from their husbands [13,17].

Ethiopia, like most other countries in Sub-Saharan Africa, has been experiencing severe HIV/AIDS epidemics. For instance, in a study done in Nazareth, in the Oromia region, more than 20 years ago, 26.8% of married Ethiopian women were found to be vulnerable to HIV [13]. As contributing factors responsible for HIV infection can differ by region, recent data are needed for a clearer understanding. Considering the current gap in the literature, this study aimed to investigate the vulnerability of married women to HIV infection in Metema District, northwest Ethiopia. Metema is the district (*woreda*) with the highest prevalence of HIV reported in the Amhara region and the 2009 antenatal care sentinel surveillance survey report of the Ethiopian Ministry of Health found an elevated prevalence (7.5%) of HIV infection in the District Hospital of Metema [11,27].

## Methods

**Ethics Statement:** The study was reviewed and approved by the Ethical Review Committee of the College of Health Sciences, Gondar University (CHS-SN-022-20). All participants provided written informed consent, and the study was conducted according to the Declaration of Helsinki.

### Study design

A community-based cross-sectional survey was done, and this study adhered to the STROBE (https://www.strobe-statement.org/) reporting guidelines.

### Setting and samples

The study was conducted in four out of the 19 administrative *kebele*s of Metema District within the Amhara region in northwest Ethiopia [28]. The inclusion criteria were married women aged ≥18 years residing in Metema District with their husbands, from four *kebele*s randomly selected by a simple randomization table. The exclusion criteria were those who were seriously ill and unable to respond to the questions, those who were unable to hear, those who had resided in the *kebele* for less than 6 months, and women who were living with HIV/AIDS. The sample size was determined using the formula for a single population proportion, considering the following assumptions: Zα/2=1.96 with a 95% confidence interval (CI), P=26.8% (prevalence of married women vulnerable to HIV based on a previous study conducted in Nazareth, Ethiopia [13]), d=0.05, design effect=2, and nonresponse rate=10%. The required sample size was 662. Of the 662 participants recruited, 657 (99.2%) responded and were included in the data analysis.

#### Measurements

A binary structured questionnaire was developed based on the literature by investigators in English, translated into the local language (Amharic) by a bilingual translator, and then back-translated to English by another bilingual translator. The internal consistency of the translation validity test was found to be adequate (0.86). The questionnaire consisted of vulnerability to HIV infection (i.e., either experiencing STI symptoms or having a history of an extramarital sexual relationship by either spouse in the past 12 months), sociodemographic characteristics, marital characteristics (marital willingness, premarital sex, frequency of being away from home), condom use with their partner (use, whether they would recommend condoms, counseling and testing, worried about being infected with HIV), HIV risk perception (whether the participant received voluntary HIV testing, worried about HIV, or worried about transmission to the fetus), and sexual communications with their partner (discussing the risk of HIV, sexual negotiating).

### Data collection

Individual households in the selected *kebele* administration were selected using a systematic random sampling technique, and the number of households sampled from the selected *kebele* administrations was determined using the proportionate to population size method. The study subjects (married women) in the selected households were interviewed by a trained assistant (two female nurse supervisors and 10 health extension workers who received 2 days of intensive training) with the questionnaire. For households with more than one married woman, only one woman was selected using the lottery method. If no one responded at a selected household that was known to contain eligible women for the study, the interviewers revisited the household three times at different time intervals; when subsequent attempts failed, the household was registered as nonresponding.

### Data analysis

The data were checked for completeness, cleaned manually, entered into statistical software for epidemiology (Epi Info ver. 7), and then exported to IBM SPSS for Windows ver. 20.0 (IBM Corp., Armonk, NY, USA) for further analysis. Frequencies and cross-tabulations were used to summarize the descriptive statistics of the data, and tables were used for data presentation. A bivariate analysis was first conducted to check which variables fulfill the minimum requirement with the dependent variable individually. Variables found to have the (*p*<.25) with the dependent variables were then entered into multiple logistic regression to control for the possible effect of confounders, and finally the variables with significant associations were identified based on odds ratios, with 95% CIs and *p*-values. A variable with a *p*-value of less than 0.05 in the multivariate analysis was considered significant.

## Results

### Sociodemographic characteristics of study participants

The mean age of the 657 respondents was 33.70±9.50 years. The Amhara ethnicity predominated (n=514, 78.2%), followed by the Gumuz ethnic group (n=78, 11.9%), which reflects the region’s ethnic composition. Four hundred and one of the participants (61.0%) were orthodox Christians.

More than half of the respondents were unable to read and write (n=377, 57.4%) and lived in a rural area (n=385, 58.6%). Four hundred and sixty-seven (71.1%) were housewives, and their average monthly income was US dollar (USD) 13.71±9.40, which was poorer than the average monthly income of the area during the study, which was USD 55.5 [28]. Three-fourths of the participants (n=493, 75.0%) reported financial scarcity affecting their ability to cover their daily living expenses (Table 1).

### Marital characteristics of the respondents

The mean duration of marriage was 12.50±8.70 years, while one-fourth (n=169, 25.7%) had been married for less than 5 years. More than half (n=353, 53.7%) got married before the age of 18 years and three-fourths (74.7%) were married according to their will. The main reason for marriage other than love was the intention to be supported financially (65.9%) (Table 2). Of the 166 women (25.3%) who were married against their will, the major reasons were as follows: forced by parents (n=101, 60.8%), pressure from relatives (n=46, 27.7%), pressure from their spouse or fiancé (n=17, 10.2%), and some sort of abduction (n=2, 1.2%). Half of the participants (n=331, 50.4%) reported never leaving their homes throughout the year, while 36.4% (n=239) could occasionally be away from home. Absence of their husband from home once per week was reported by 357 participants (54.3%), and absence once per month was reported by 210 (32.0%). Premarital sexual relationships were reported for 241 of women (36.7%) and 140 of the husbands (21.3%) (Table 2).

### Condom use and human immunodeficiency virus risk perception

Of all respondents, 71 (10.8%) had used a condom with their husbands in the past 12 months and 46 (64.8%) used condoms regularly. More than half of the respondents (n=386, 58.8%), however, did not recommend condom use in married couples. The main reason condoms were not recommended for couples (n=314, 81.3%) was that they could cause offense by implying speculation or suspicion that the husband has HIV or another STI.

Regarding HIV counseling and testing, 361 participants (54.9%) had received HIV counseling and testing before they got married and 224 (34.1%) had done so in the past 12 months preceding the study period.

In response to items on risk perceptions of HIV infection, 204 women (31.1%) reported that they had ever worried about being infected by HIV, and of those, 171 (83.8%) had such worries in the last 12 months. The main reasons for their worries were their husbands’ infidelity (43.8%) and their inability to be sure about their husbands’ serostatus (30.9%).

Finally, 103 women (15.7%) had been pregnant in the last 12 months, and most of them (n=79, 76.7%) feared HIV transmission to their fetuses (Table 3).

### Sexual communication within married couples

Less than one-fifth of the married women (n=124, 18.9%) communicated about sexual matters with their husbands. Of this number, three-fourths (n=93, 75.0%) reported that the discussions were initiated by their husbands, and nearly two-thirds (n=81, 65.3%) encountered disagreement during the discussions. Of the respondents who communicated about sexual matters, more than half 73 of the couples (58.9%) trusted each other, which encouraged the transparency of their discussions and sexual negotiations (Table 4).

### Vulnerability to human immunodeficiency virus infection

Of the 657 respondents, vulnerability to HIV as measured by either experiencing STI symptoms or having a history of an extramarital sexual relationship by either spouse in the past 12 months was found in 148 married women (22.5%), with STI symptoms reported for 47 women (7.2%) and 60 husbands (9.1%). Participants reported extramarital sexual affairs in the past 12 months for 39 women (5.9%) and 93 of their husbands (14.2%).

### Factors associated with vulnerability to human immunodeficiency virus infection

Based on the bivariate analysis result (*p*<.25), candidate predictable variables for multivariable logistic regression analysis were as follows: residence, many children, income insufficiency, marriage willingness, condom use in married couples, extramarital relationship, sex before marriage, discussions about sexual matters, worrying about being infected by HIV, deciding on sexual matters by negotiation, and having experienced pregnancy in the last 12 months.

As reported in Table 5, women who did not discuss the risk of HIV infection with their husbands had fivefold odds of being vulnerable to HIV than those who did (adjusted odds ratio [AOR], 5.02; 95% CI, 1.43–17.5). Women who had engaged in premarital sex had 80% decreased odds (AOR, 0.20; 95% CI, 0.05–0.77); those who were not worried about being infected by HIV had 73% decreased odds (AOR, 0.27; 95% CI 0.08–0.94); those with sufficient income had 44% decreased odds (AOR, 0.56; 95% CI, 0.16–0.86); and women with <4 children had 31% decreased odds (AOR, 0.69; 95% CI, 0.50–0.97) of vulnerability to HIV compared to their counterparts.

## Discussion

Studies assessing married women’s vulnerability to HIV infection are lacking, and to our knowledge, this is the second study in Ethiopia to focus on this topic. The goal of this study was to assess the vulnerability to HIV infection and associated factors in married women in Metema District, Ethiopia. We found that 22.5% of participants were vulnerable to HIV, as determined by either having a symptom of an STI or a history of an extramarital sexual relationship by either spouse in the past 12 months.

This finding is lower than that of the previous study conducted in Nazareth, in central Ethiopia, which reported that 26.8% of married women were vulnerable to HIV infection [13]. A possible explanation for the discrepancy between these study results might be due to time-based differences in HIV prevalence. Nazareth was previously the area in Ethiopia with the highest prevalence of HIV, whereas now the Gambella region shows the highest prevalence, followed by the Addis Ababa administrative region and the Amhara region. In this study, 39 (5.9%) and 93 (14.2%) of married women and their husbands, respectively, had extramarital relationships within the last 12 months, as reported by married women. Another study conducted in Kenya indicated that married men engaging in sex with extramarital partners had an increased vulnerability to HIV infection [29].

In this study, married women who did not discuss the risk of HIV infection with their husbands were five times more likely to be vulnerable to HIV than those who did, which was supported by a study done in Mozambique [30]. In a qualitative study conducted in Nigeria, women who fully engaged in open discussions about sexual health became knowledgeable about sex, and this ultimately improved women’s ability to make informed decisions about risk reduction. On a similar note, research conducted in Malawi revealed that women who had open discussions with their husbands were less likely to be vulnerable to HIV [24]. The finding of the present study in which only 18.9% reported sexual communication with their husband, is similar to a study conducted in Nepal that showed that nearly half of the participants not being able to ask their husbands about HIV and other STIs even if they wondered about being vulnerable to HIV [31]. A reason for this might be that women are financially dependent on their husbands and unable to make independent decisions because of male dominance. As such, this underscores the importance of helping married couples to communicate about sex, especially in relation to the risk of HIV and other STIs.

Women who did not have sex before marriage had 80% decreased odds of vulnerability to HIV infection than those who had premarital sex. Of those who had premarital sex, 7.5% used condoms regularly on all occasions. This is quite different from the study conducted in Nazareth, Ethiopia, in which 33.9% of married women stated that they had engaged in premarital sex, and of those, 13.9% reported having used condoms consistently on all occasions [13]. Variations in condom utilization during premarital sex might be due to differences in educational status, culture, and ability to access information. Nazareth, which is located in the center of Ethiopia, has a higher degree of educational accessibility, which changes the culture of gender-based differences. Despite the over 20-year gap of this study, male dominance is still highly prevalent in Metema District.

Regarding the finding that women not worried about being infected by HIV were 73% less likely to be vulnerable than those who were worried, it is worthy to the main reasons for their worries; i.e., husbands’ infidelity (43.8%) and their inability to be sure about their husbands’ serostatus (30.9%). This is supported by previous research conducted in Uganda, Nigeria, Ethiopia (Nazareth), and Mozambique [2,13,32]. This suggests areas for interventional health studies.

As for the study finding that married women who had sufficient income to cover their expenses were 44% less likely to be vulnerable than those who had insufficient income, this indicates the commonality of economic factors that increase the vulnerability of women to HIV infection in developing countries [2,20,31]. Women who depend on their husbands for financial security are likely to be uneducated, and thus likely to lack knowledge of the consequences of unsafe sex practices. Women who married to secure their financial needs and those who were married against their will were also found to be more vulnerable to HIV infection and more likely to be unaware of being at risk [13]. In many societies, especially in developing countries, women and girls are the primary victims of poverty. Of the 1.2 billion people living on less than USD 1 a day, 70% are women. Women’s economic dependence also makes them vulnerable to HIV/AIDS [23]. Thus, legal considerations, political involvement, and economic stability may help reduce women’s vulnerability to HIV infection.

Finally, this study found that women who had three or fewer children were 31% less likely to be vulnerable to HIV infection than those with four or more children. Number of children may reflect level of income in this sample, as the overall sample reported financial difficulty and sufficient income was an influential factor as noted above. However, the lack of similar studies on this topic makes it difficult to compare the findings.

A limitation of this study is that despite efforts to randomly select from the community, the attitudes of married women toward their sexual partners, as well as the sexual history of married men reported by their wives, might have been either under-reported or over-reported. Although more than half of the participants were rural inhabitants and unable to read and write, responding through a research assistant provided them with the opportunity to participate.

In conclusion, this study found that vulnerability to HIV, as measured by either experiencing STI symptoms or having a history of an extramarital sexual relationship by either spouse in the past 12 months, was found in 148 married women (22.5%). As such, to reduce married Ethiopian women’s vulnerability to HIV infection, efforts to encourage negotiation about sexual matters and communication about HIV infection within married couples are crucial. Particular attention is also needed for women with a history of premarital sex, express worry about HIV infection, lack sufficient income, and have a higher number of children (≥4).

Since the health of reproductive women is one of the top priorities of the government, empowering and economically strengthening married women through education is important. The Ethiopian Federal Ministry of Health and the Amhara regional health bureau should collaborate to provide continued training for health extension workers to address married couples through health education, especially focusing on clearer sexual communication and risk reduction measures.

## Figures and Tables

**Table 1.** Sociodemographic characteristics of married women in Metema District, Amhara region, Ethiopia in 2020 (N=657)

Variable	Categories	n (%)
Age (year)	(mean±SD, 33.70±9.50)	
	18–24	103 (15.7)
	25–29	133 (20.2)
	30–34	125 (19.0)
	35–39	124 (18.9)
	>40	172 (26.2)
Ethnic group	Amhara	514 (78.2)
	Gumuz	78 (11.9)
	Tigre	43 (6.5)
	Oromo	22 (3.3)
Religion	Orthodox Christian	401 (61.0)
	Muslim	233 (35.5)
	Protestant	15 (2.3)
	Catholic	8 (1.2)
Educational level	Unable to read and write	377 (57.4)
	Grade 1–8	158 (24.0)
	Grade 9–10	83 (12.6)
	Grade 11–12	13 (2.0)
	Above grade 12	26 (4.0)
Current occupation	Housewife	467 (71.0)
	Farmer	67 (10.2)
	Private business	42 (6.4)
	Governmental employee	36 (5.5)
	NGO	34 (5.2)
	Daily laborer	11 (1.7)
Women with sufficient income	Yes	164 (25.0)
	No	493 (75.0)
Number of children	(median, 4; range, 0–7)	
	Yes	563 (85.7)
	No	94 (14.3)
Place of residence	Urban	272 (41.4)
	Rural	385 (58.6)

NGO: Nongovernmental organization.

**Table 2.** Marital characteristics of married women in Metema District, Amhara region, northwest Ethiopia in 2020 (N=657)

Variable	Categories	n (%)
Duration of marriage (year)	<5	169 (25.7)
	5–9	95 (14.5)
	10–14	137 (20.9)
	15–19	96 (14.6)
	>20	160 (24.4)
Age at first marriage (year)	<18	353 (53.7)
	≥18	304 (46.3)
Marriage willingness	No	166 (25.3)
	Yes	491(74.7)
Husband’s premarital sex	No	517 (78.7)
	Yes	140 (21.3)
Wife’s premarital sex	No	416 (63.3)
	Yes	241 (36.7)
(If yes) Marriage for other than love	No	324 (65.9)
	Yes	167 (33.8)
(If yes) Reason for marriage	Unintended pregnancy	32 (19.2)
	To be supported financially	110 (65.9)
	Academic failure	25 (14.9)
Frequency of wife being away from home	At least once per week	55 (8.4)
	At least once per 3 months	8 (1.2)
	At least once per 6 months	24 (3.7)
	Occasionally	239 (36.4)
	Never	331 (50.4)
Frequency of husband being away from home	At least once per week	357 (54.3)
	At least once per month	210 (32.0)
	At least once per 3 months	10 (1.5)
	At least once in 6 months	49 (7.4)
	Never	31 (4.7)

**Table 3.** Condom use and HIV risk perceptions among married women in Metema District, Amhara region, northwest Ethiopia in 2020 (N=657)

Variable	Categories	n (%)
Used condom with husband in the past 12 months	No	586 (89.2)
	Yes	71 (10.8)
(If yes) Used condom regularly with husband	Regular use	46 (64.8)
	Non-regular use	25 (35.2)
(If yes) Reason for using condoms	To prevent pregnancy	56 (78.9)
	To be protected from STI and HIV	15 (21.1)
Would recommend condom for married couples	No	386 (58.8)
	Yes	271 (41.2)
(If no) Reason for not recommending condoms	Inconvenient to use	31 (8.0)
	Could offend one’s husband	314 (81.3)
	Other methods are easily available	41 (10.6)
Voluntary premarital HIV counseling and testing	No	296 (45.1)
	Yes	361 (54.9)
Voluntary HIV counseling and testing in the past 12 months	No	433 (65.9)
	Yes	224 (34.1)
Ever worried about being infected with HIV	No	453 (68.9)
	Yes	204 (31.1)
Worry in the past 12 months (from the 204 “yes” respondents above)	No	33 (16.2)
	Yes	171 (83.3)
(If yes) Reason for worrying about HIV infection	Not sure of husband’s serostatus	53 (31.0)
	Not sure if husband is faithful	75 (43.9)
	Not sure of own serostatus	25 (14.6)
	Injured by sharp materials	18 (10.5)
Pregnant in the last 12 months	No	554 (84.3)
	Yes	103 (15.7)
(If yes) Worried about transmission to fetus	No	24 (23.3)
	Yes	79 (76.7)

HIV: Human immunodeficiency virus; STI: sexually transmitted infection.

**Table 4.** Sexual communication within married couples in Metema District, Amhara region, Ethiopia in 2020 (N=657)

Variable	Categories	n (%)
Communication about sexual matters with husband	No	533 (81.1)
	Yes	124 (18.9)
(If yes) Person who initiates discussion on sexual matters	Husband	93 (75.0)
	Wife (self)	14 (11.3)
	Both	17 (13.7)
(If yes on sexual communication) Discussed and disagreed on sexual matters	No	43 (34.7)
	Yes	81 (65.3)
(If yes) Frequency of conflict	Always	12 (14.8)
	Usually	40 (49.4)
	Occasionally	29 (35.8)
(If yes on disagreement) Reaching conflict resolution	Always	16 (19.8)
	Most of the time	29 (35.8)
	Occasionally	24 (29.6)
	Never	12 (14.8)
(If yes on sexual communication) Discussed family planning	No	76 (61.3)
	Yes	48 (38.7)
(If yes on sexual communication) Discussed risk of HIV/AIDS	No	81 (65.3)
	Yes	43 (34.7)
(If yes on sexual communication) Discussed trustfulness of marriage	No	51 (41.1)
	Yes	73 (58.9)
Decided sexual matters by negotiation	No	586 (89.2)
	Yes	71 (10.8)
Forced sexual practice by husband	No	406 (61.8)
	Yes	251 (38.2)

AIDS: Acquired immune deficiency syndrome; HIV: human immunodeficiency virus.

**Table 5.** Factors associated with the vulnerability of married women to HIV infection in Metema District, Amhara region, Ethiopia in 2020 (N=657)

Explanatory variable	Categories	Vulnerability to HIV, n (%)		Crude OR (95% CI)	Adjusted OR (95% CI)
		Yes (n=148)	No (n=509)		
*Sociodemographic factors*					
Number of children	≥4	148 (42.0)	204 (58.0)	1.07 (1.01–1.7)^*^	0.69 (0.50–0.97)^*^
	<4	123 (40.3)	182 (59.7)	1	1
Residence	Urban	47 (17.3)	225 (82.7)	1.70 (0.86–2.86)	0.64 (0.21–1.97)
	Rural	101 (26.2)	284 (73.8)	1	1
Sufficient income to cover expenses	No	107 (21.7)	386 (78.3)	0.83 (0.55–0.25)	0.56 (0.16–0.86)^*^
	Yes	41 (25.0)	123 (75.0)	1	1
*Marital characteristics*					
Marriage willingness	No	46 (27.9)	119 (72.1)	1.47 (0.98–2.21)	0.45 (0.13–1.54)
	Yes	102 (20.7)	390 (79.3)	1	1
Married before	No	85 (19.7)	346 (80.3)	0.63 (0.43–0.92)	1.68 (0.55–5.08)
	Yes	63 (27.9)	163 (72.1)	1	1
Husband’s premarital sex	No	96 (18.6)	421 (81.4)	0.38 (0.25–0.58)	0.75 (0.20–2.85)
	Yes	52 (37.1)	88 (62.9)	1	1
Wife’s premarital sex	No	54 (13.0)	362 (87.0)	0.23 (0.16–0.34.)	0.20 (0.05–0.77)^**^
	Yes	94 (39.0)	147 (61.0)	1	1
*Condom use and HIV risk perception factors*					
Used a condom with husband	No	122 (20.8)	464 (79.2)	0.45 (0.27–0.76)	0.88 (0.24–3.27)
	Yes	26 (36.6)	45 (63.4)	1	1
Worried about being infected by HIV	No	63 (13.9)	391 (86.1)	0.22 (0.15–0.32)	0.27 (0.08–0.94)^***^
	Yes	85 (41.9)	118 (58.1)	1	1
Pregnancy in the last 12 months	No	112 (20.2)	443 (79.8)	0.46 (0.29–0.73)	2.0 (0.29–13.6)
	Yes	36 (35.3)	66 (64.7)	1	1
*Sexual communication factors*					
Discussed the risk of HIV infection	No	27 (33.3)	54 (66.7)	3.08 (1.15–8.20)	5.02 (1.43–17.5)^*^
	Yes	6 (14.0)	37 (86.0)	1	1
Decided on sexual matters by negotiating	No	122 (20.8)	464 (79.2)	0.45 (0.27–0.76)	0.82 (0.18–3.64)
	Yes	26 (36.6)	45 (63.4)	1	1

CI: Confidence interval; HIV: human immunodeficiency virus; OR: odds ratio.

* *p*<.050,

** *p*<.001,

*** *p*<.0001.
